# Effect of Temperature Pre-Exposure on the Locomotion and Chemotaxis of *C. elegans*

**DOI:** 10.1371/journal.pone.0111342

**Published:** 2014-10-31

**Authors:** Lipika Parida, Sudarsan Neogi, Venkat Padmanabhan

**Affiliations:** Department of Chemical Engineering, Indian Institute of Technology, Kharagpur, West Bengal, India; CSIR-Central Drug Research Institute, India

## Abstract

The effect of temperature pre-exposure on locomotion and chemotaxis of the soil-dwelling nematode *Caenorhabditis elegans* has been extensively studied. The behavior of *C. elegans* was quantified using a simple harmonic curvature-based model. Animals showed increased levels of activity, compared to control worms, immediately after pre-exposure to 30*°*C. This high level of activity in *C. elegans* translated into frequent turns by making ‘complex’ shapes, higher velocity of locomotion, and higher chemotaxis index (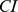) in presence of a gradient of chemoattractant. The effect of pre-exposure was observed to be persistent for about 20 minutes after which the behavior (including velocity and 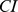) appeared to be comparable to that of control animals (maintained at 20*°*C). Surprisingly, after 30 minutes of recovery, the behavior of *C. elegans* continued to deteriorate further below that of control worms with a drastic reduction in the curvature of the worms' body. A majority of these worms also showed negative chemotaxis index indicating a loss in their chemotaxis ability.

## Introduction

The nematode *Caenorhabditis elegans* is a well-studied model organism whose neuronal network and genomic sequence have been thoroughly established [1]–[3]. The complete description of the morphology and synaptic connectivity of all 302 neurons in *C. elegans* has raised the prospect of analyzing and understanding its complex behavioral traits. Like all animals, *C. elegans* also respond to various environmental cues such as chemical and thermal stimuli by detecting and processing the sensory information within its nervous system. The soil-dwelling nematode feeds on bacteria [4] and shows strong chemotactic response to chemicals such as water-soluble salts (e.g., NaCl and lysine), volatile odorants (e.g., benzaldehyde and isoamyl alcohol) and amino acids [5]–[7]. The highly developed chemosensory system in these nematodes is used not only to detect food but also danger and make appropriate decisions to effectively avoid it. Such an implementation of advanced locomotory behavior with only 302 neurons is extremely exceptional.

The nematode typically moves forward on either of its sides by producing an undulatory motion along the length of its body. Despite the simplicity of its body shape and motor control, *C. elegans* can move efficiently in complex multi-phase environments. Several attempts to classify the locomotory behavior of *C. elegans* have been made and different mechanisms have been proposed [8]. General aspects of undulatory worm propulsion were reported more than half a century ago [9], [10]. According to the classical picture, the undulatory locomotion of *C. elegans* relies on the anisotropy of the transverse and lateral friction between the worm body and the surrounding medium. If the transverse friction coefficient, 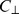 (perpendicular to the worm body curvature) is much larger than the lateral friction coefficient 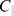, the worm moves forward efficiently. The sensory neurons detect attractants/repellents and direct the worm accordingly to positive or negative chemotaxis behaviors [5]–[7], [11], [12]. In absence of any chemical stimulus, *C. elegans* exhibits two distinct behaviors; one in which it moves rapidly and reverses frequently while trying to identify any source of bacteria. This strategy is called pivoting, area-restricted search, or local search [13]–[15] that is similar to the basic run-pirouette mechanism described by Pierce-Shimmomura *et al.* [16]. Upon failure to find and locate food source, *C. elegans* switches to the second behavioral state where reversals are suppressed and the nematode travels longer distances without significant changes in the direction of motion [13], [15]. *C. elegans* can also learn and remember the stimuli it encounters. They show non-associative forms of learning, such as habituation and dishabituation, as well as associative forms of learning, such as classical conditioning and differential classical conditioning [5], [17]–[19].

Several studies have been reported on the behavior of *C. elegans* on exposure to various environmental conditions [20]–[23]. For example, the chemotactic response of *C. elegans* to Sodium Chloride was enhanced when the animals were maintained in presence of Sodium Chloride with food bacteria. Whereas, it was suppressed when *C. elegans* were maintained in presence of Sodium Chloride without food [24]. In *C. elegans*, continuous presentation of chemical and mechanical stimuli can cause a decrease in the level of response to the same stimuli [7], [25]–[27]. Recently, Matsuura *et al.* [28] showed that the chemotactic behavior of *C. elegans* to Sodium Acetate was significantly enhanced when the animals were pre-exposed to the chemical for 90 minutes. This increase was observed for about 6 hours after pre-exposure but not after 12 hours. All of the above studies were performed at room temperature, particularly in experimental laboratories that were probably temperature-controlled. However, temperature fluctuations in the wild are significantly higher and may have a strong impact on the locomotion and chemotaxis of *C. elegans*. They may have to adapt their life cycle depending on the environmental conditions of changing seasons through out the year. So, it is crucial to understand the effect of temperature on such behaviors of *C. elegans*. This is the motivation of the present study. A similar study on the fresh-water snail *Lymnaea stagnali* found that its locomotion and respiration increased with an increase in temperature that may be a result of temperature-dependent reactions in its neurons [29].

*C. elegans* thrive in environments where the temperature ranges from 15–25*°C*. A recent study by MacMillan *et al.* [30] found that an increase in culture temperature increases the speed and wavelength of the nematode. In this work, we focus on the behavioral changes in *C. elegans* when they are pre-exposed to temperatures higher than the widely used temperature of 20*°*C and study its effect on the locomotion and chemotaxis. We found that, *C. elegans* pre-exposed to higher temperature showed increased levels of activity, speed, and chemotaxis initially. But, surprisingly after 30 minutes of recovery period at room temperature, the trend is completely reversed. The aim of this study is to determine the effect of high temperature exposure on *C. elegans*' behavior, how long the retention time of enhancement of chemotactic response continues after pre-exposure, and to understand the behavioral changes *C. elegans* adopts in response to such environmental changes.

## Results

### Comparison of shapes between pre-exposed and control animals

**Figure 1:**
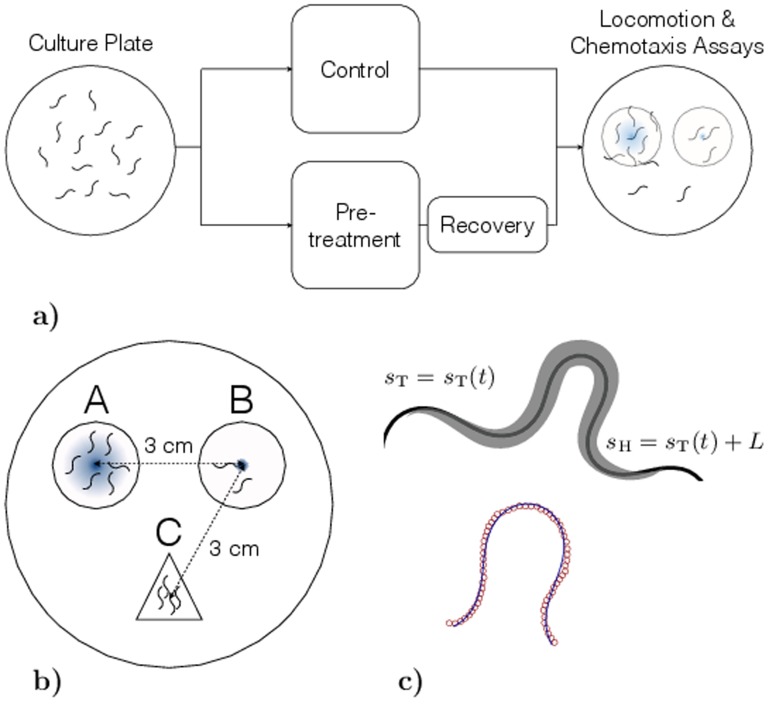
Methods. a) Experimental procedure b) Schematic of chemotaxis assay plate. The locations A and B represent chemoattractant and control spots, respectively. The circles indicate a region with a diameter of 3 cm around the compounds that was used to determine the chemotaxis index. The location C represents the spot at which *C. elegans* were initially placed and c) Schematic of the harmonic-curvature model that shows skeleton data for worm superimposed with the fit.

To understand the effect of temperature pre-exposure on the behavior of *C. elegans*, we performed a series of experiments on two sets of worms that were cultured at 20*°*C and maintained at similar dietary conditions. One of the sets was then pre-exposed to a temperature of 30*°*C for about 30 minutes just before the experiments, while the other was maintained at 20*°*C. After pre-exposure, images of worms were extracted from movies that were recorded as per the recovery times mentioned in the Materials and Methods section. The set of worm cultures maintained at 20*°*C was used to perform control experiments for comparison as shown in Fig. 1a.

**Figure 2:**
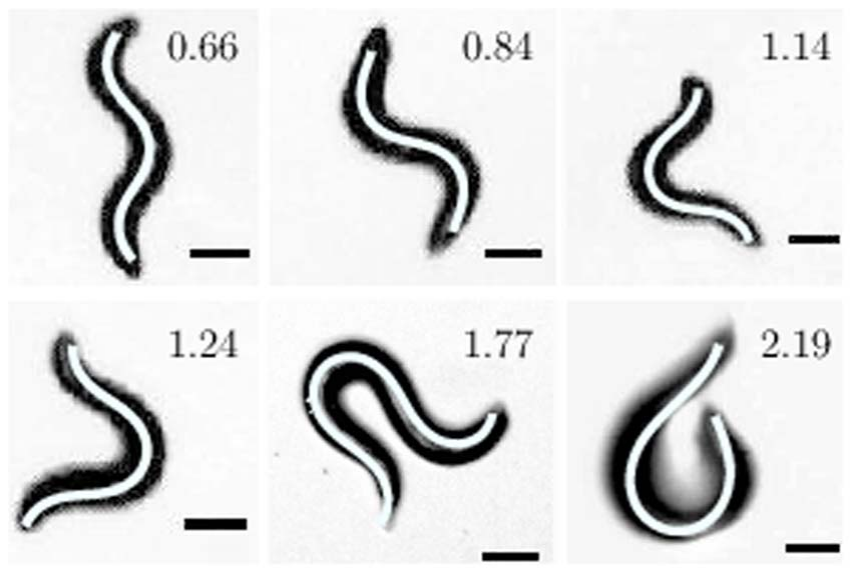
Various shapes of crawling *C. elegans*. The corresponding model representations are shown as white lines along the backbone of worms. The experimental images were obtained at 20*°*C on 2 wt% agar. The ratio 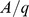 for all the shapes are also shown.

The behavior of *C. elegans* can be quantified by analyzing the shape of their body during crawling motion. In general, it is known that *C. elegans* travel forward by making undulatory movements along the length of their body and make sharp turns by curving their body such that it resembles an omega or a loop shape. The level of activity in *C. elegans* can be directly associated with the number of sharp turns they make in a given time as it requires the animal to increase its instantaneous velocity to accomplish such a turn [16]. We analyzed and compared the shapes of pre-exposed and control animals using the curvature-based model, described in the Materials and Methods section, to extract the parameters, 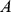 and 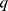 for the curvature of the worm's body. Fig. 2 shows the various shapes *C. elegans* make in absence of food. The corresponding 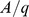 values are also shown. The values of curvature parameters, 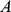 and 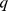 are dimensionless as fitting was done on the normalized skeleton data. we note that it is possible to classify the worm body shapes as ‘regular’ (where the animals exhibit a sine-shaped curve) when 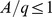 and ‘complex’ (omega, loops, etc.) when 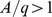.

**Figure 3:**
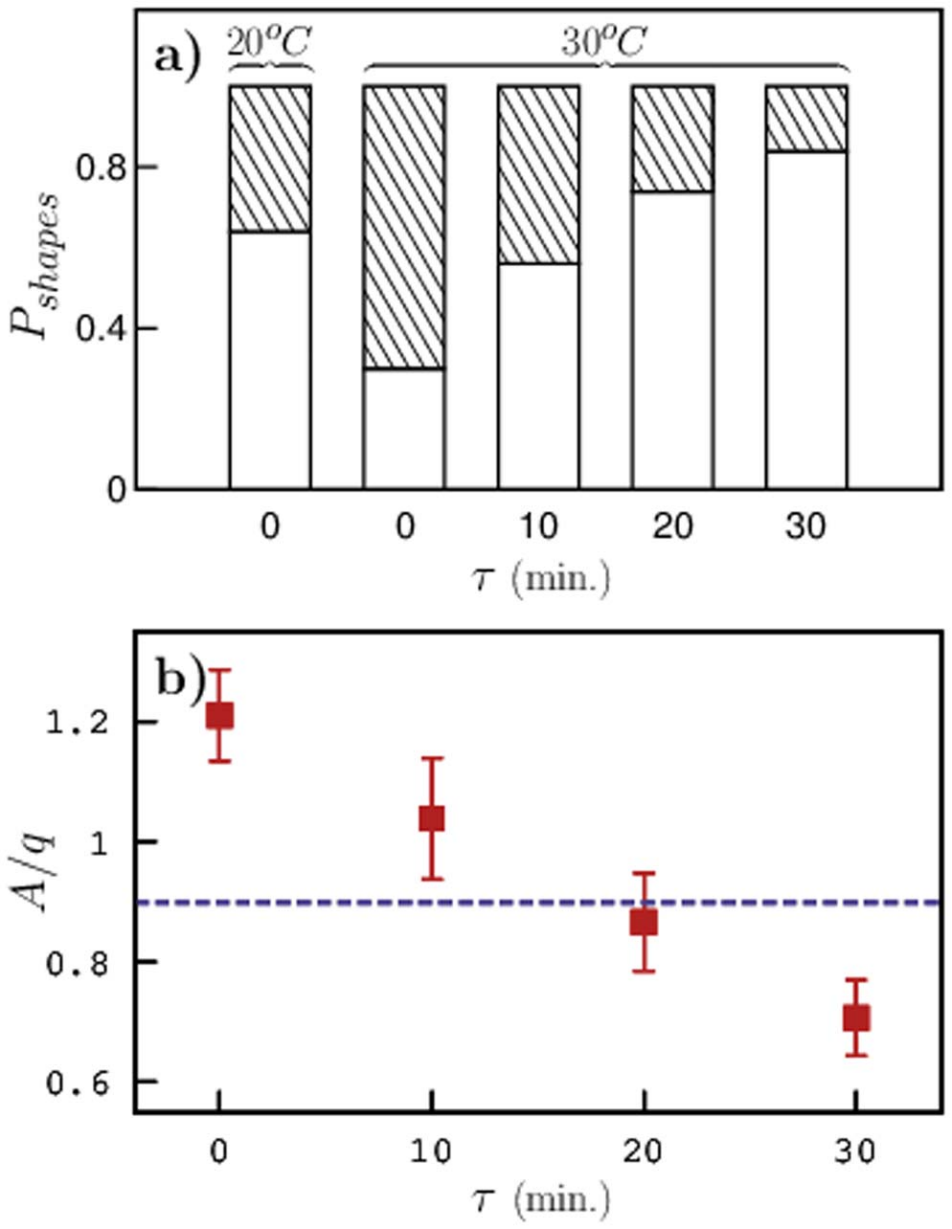
Classification of worm shapes. a) Probability of shapes of *C. elegans* classified into ‘regular’ (open) and ‘complex’ (filled) based on the average ratio 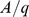. The corresponding temperature is shown above the bars and b) average ratio of parameters 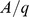 for pre-exposed worms as a function of recovery time. The average ratio for control animals is shown as a dashed line.

Several images of worms from both sets (control and pre-exposed) were analyzed to extract the average ratio of parameters, 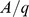. The shapes of *C. elegans* were then classified as either ‘regular’ or ‘complex’ based on the values of 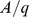. This procedure was also repeated for pre-exposed animals that were allowed to recover from high-temperature shock, in which case, images were captured after the prescribed recovery period. Fig. 3a shows the probability of worm shapes for all cases. The average probability was obtained from 20 independent experiments for each set (containing ∼ 400 worms). Control animals that were maintained at a temperature of 20*°*C were observed to be mostly ‘regular’ with only about 30% of them forming omegas and loops indicating a normal behavior. On the other hand, animals that were pre-exposed to 30*°*C showed increased levels of activity with at least 70% of them forming complex shapes immediately after pre-exposure. As the worms were allowed to recover from the high-temperature shock, we observed a gradual decrease in the curvature of the worms' body and the probability of worms making complex shapes also decreased.

Although, it is evident from Fig. 3a that pre-exposing the worms to high temperature has a significant impact on their body shapes, to understand how long *C. elegans* retain this effect, we plotted the average ratio of parameters 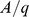 of the worm body shapes in Fig. 3b. The average ratio 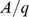 of the pre-exposed worms is significantly higher than that of control animals and as the worms were allowed to recover, 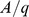 decreases as expected and becomes comparable to that of control animals after about 20 minutes of recovery. Surprisingly, after 30 minutes of recovery, the average 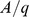 is lower than that of control animals indicating a drop in the worm's body curvature. The shapes of these worms were almost straight with mild undulations along the length of their body and they moved very slowly. We are currently investigating worms that were allowed to recover for more than 30 minutes after pre-exposure.

### Locomotion of pre-exposed animals

**Figure 4:**
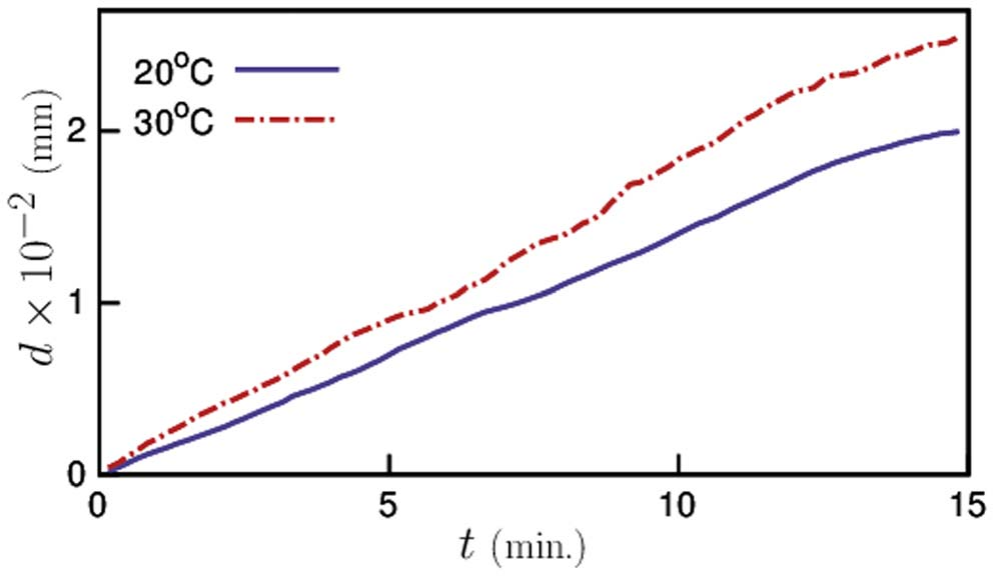
Root mean squared distance traveled by the worms as a function of time. The pre-exposed worms traveled larger distances in the 15 minute window of observation.

To study changes in the locomotory behavior of *C. elegans* when exposed to temperatures higher than their culture temperature, we plotted the root mean squared distance (RMSD) traveled by the worms as a function of time for both temperatures in Fig. 4. We note that at any given time the distance traveled by animals that were pre-exposed was higher than that of control animals. This indicates that pre-exposed worms were not only more active but also moved faster with higher velocity than control animals. The average velocities of the two sets of animals, calculated from slope of the RMSD plot, are given in Table 1. The average velocity of worms from the control experiments was found to be in excellent agreement with previous studies reported at that particular temperature [31], [32]. As the pre-exposed animals moved faster and traveled greater distances (in a given time) than the control animals, one might expect that they moved from one place to another with greater efficiency. On the contrary, the net displacement of pre-exposed animals from their respective starting points has been consistently smaller than those of the control animals (Table 1).

**Table 1:** Average velocity, calculated from the slope of RMSD curves (Fig. 4) and the net displacement of worms from their respective starting points for control and pre-exposed animals.

Temperature *°*C	Velocity 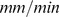	Net Displacement 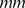
20	14.09 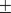 0.338	8.71 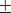 0.46
30	18.7 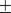 0.713	5.63 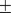 0.49

The net displacement is the distance between initial and final positions of the worms after 15 minutes.

**Figure 5:**
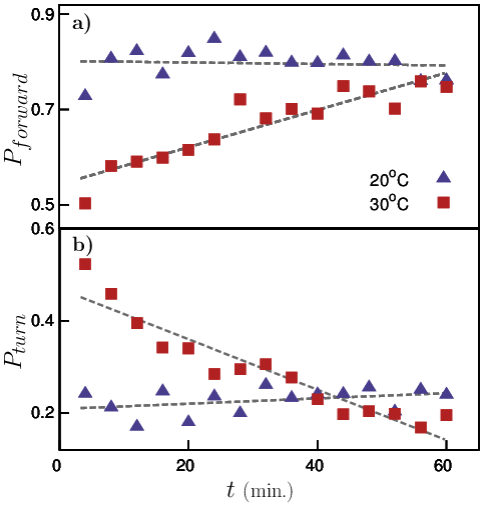
Probability of worm motion. a) travelling forward and b) turning for both pre-exposed (squares) and control (triangles) worms. The dashed-lines are just guides to the eye.

To understand, why the net displacement of pre-exposed animals were always smaller than that of control animals, we analyzed the movement of both sets of animals and found that although the pre-exposed animals moved faster as expected, they made more number of turns using ‘complex’ shapes (as indicated in Fig. 2). As a result, they wandered mostly around the same spot covering a very little effective distance from their starting points. Whereas, the control animals moved predominantly in the forward direction thus enabling them to move away from their respective starting points quickly. We quantify this observation by plotting the probability of forward motion (Fig. 5a) and the average turning probability (Fig. 5b) for both sets of worms. For these experiments, the pre-exposed animals were placed under the microscope immediately after the predefined time of pre-exposure and images were captured at regular intervals of 4 minutes. The probabilities were then calculated by counting the number of worms moving forward/turning divided by the total population of worms. The probability of worms moving in forward direction (with ‘regular’ shapes) was fairly constant for control animals, but for pre-exposed worms, the probability was very low immediately after pre-exposure indicating that very few worms moved forward and as the worms recovered from the high-temperature shock, the probability increased and eventually became comparable to that of the control animals. The average turning probability also showed that pre-exposed animals turned (using ‘complex’ shapes) more frequently than control animals immediately after exposure.

**Figure 6:**
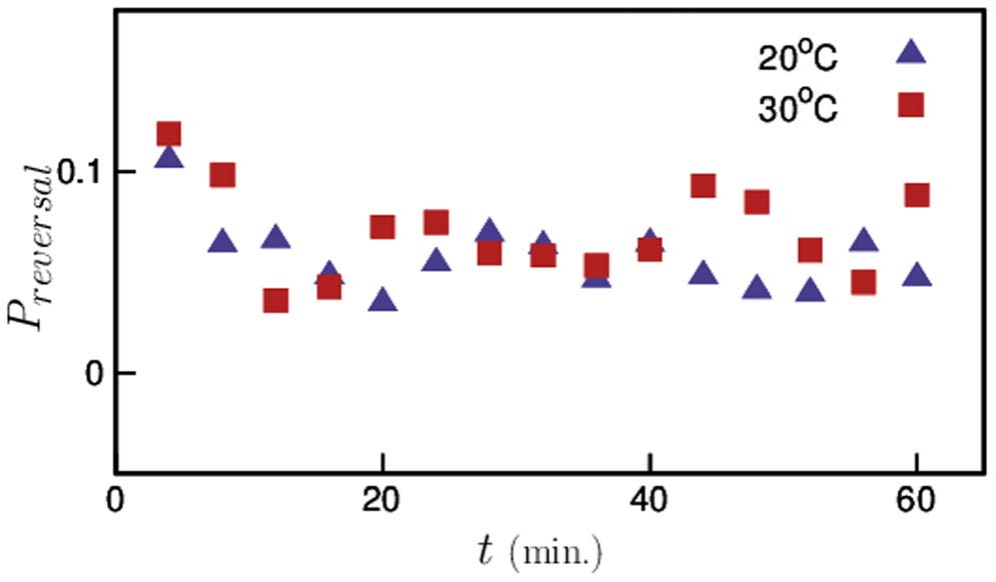
Probability of reversals for pre-exposed and control worms. No significant effect of temperature pre-exposure was found on reversals.

Next, we also looked into the effect of temperature pre-exposure on another important mode of *C. elegans* locomotion; reversals. *C. elegans* occasionally move back (tail first) retracing its original path before making a drastic change in its direction of motion. Several studies [33] have found that *C. elegans* use this technique to quickly retract from undesirable stimuli such as touch, heat, etc. Fig. 6 shows the probability of reversals for both sets of animals and we note that high-temperature pre-exposure does not have any significant effect on the reversal motion of *C. elegans*.

### Chemotaxis behavior of pre-exposed animals

**Figure 7:**
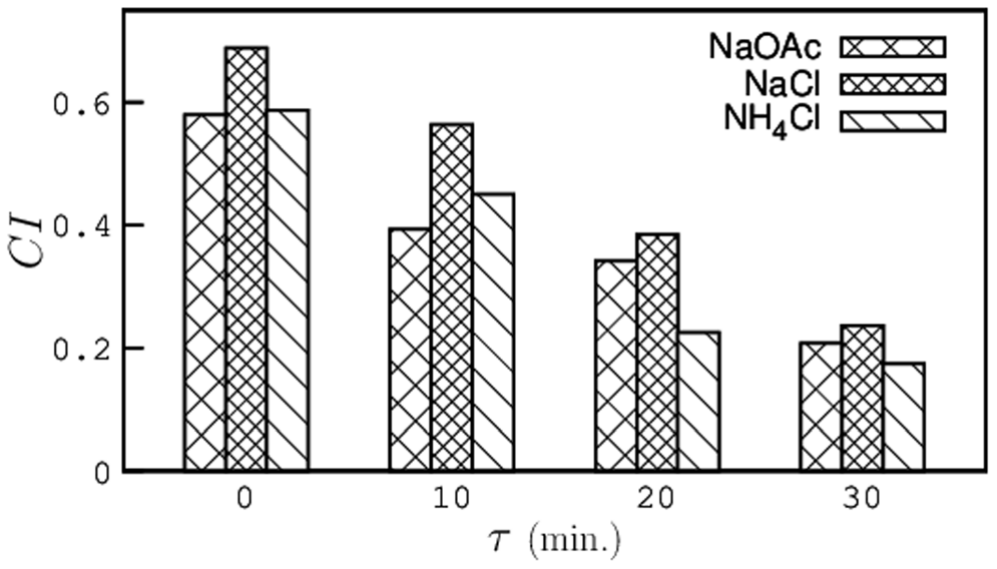
Chemotaxis Indices (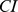) of pre-exposed worms as a function of recovery time for three different chemoattractants. The average chemotaxis indices of control animals are NaOAc  =  0.329, NaCl  =  0.352, and NH_4_Cl  =  0.356.

To study the effect of pre-exposure on the chemotaxis behavior of *C. elegans*, we preformed several experiments on both pre-exposed and control animals using three different chemoattractants (sodium acetate, sodium chloride and ammonium chloride). The chemotaxis assay plates were prepared for experiments as mentioned in the Materials and Methods section. After pre-exposure, *C. elegans* were allowed to recover from the temperature shock for 0, 10, 20, and 30 minutes at room temperature before transferring them onto the assay plate. Fig. 7 shows the chemotaxis index (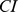) for the two sets of animals with the recovery time plotted on the x-axis. For all three chemoattractants, the chemotaxis indices for worms pre-exposed at 30*°*C were found to be significantly higher than that of control animals. As the animals were allowed to recover, the 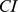 decreases and interestingly, for worms that were allowed to recover for more than 20 minutes, the 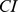 falls below that of control animals. In this case, we observed that several worms showed negative chemotaxis behavior to the chemoattractant which indicates that there is a loss in chemotaxis ability of worms that were recovered for about 30 minutes after pre-exposure.

**Figure 8:**
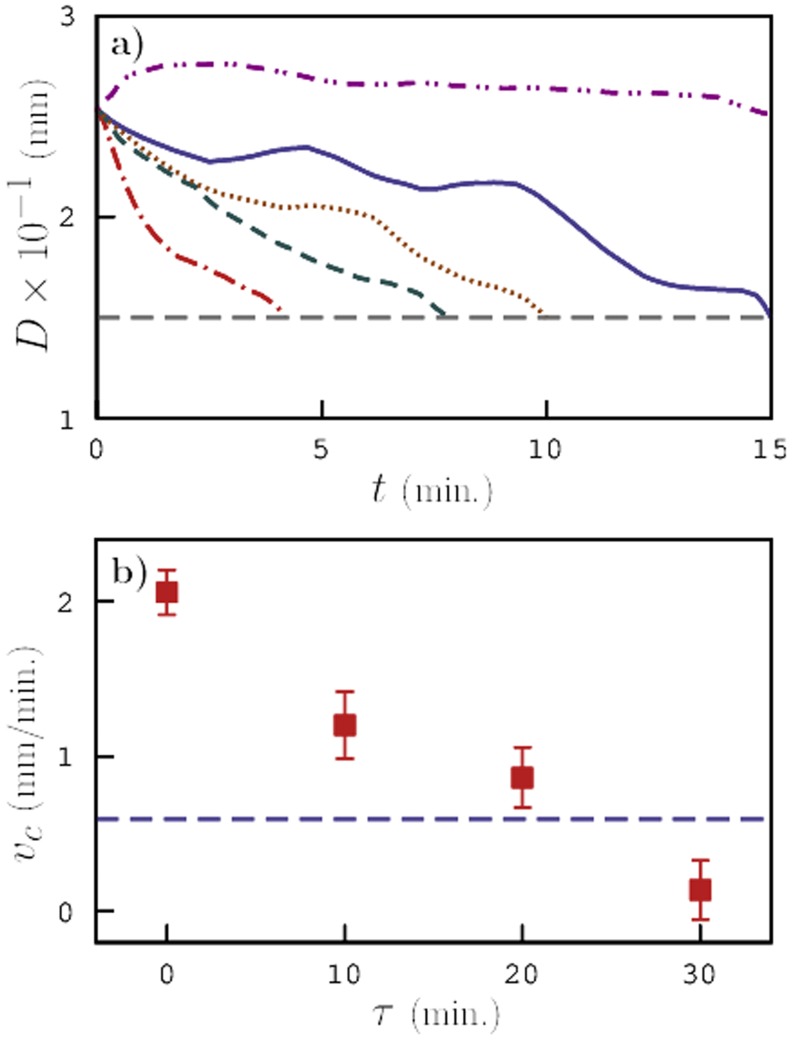
Chemotaxis velocity. a) Average distance between *C. elegans* and chemoattractant concentration peak as a function of time during the chemotaxis experiment for control worms (solid) and pre-exposed animals after 0 (dash-dot), 10 (dashed), 20 (dotted), and 30 (dash-dot-dot) minutes and b) speed with which *C. elegans* approach the concentration peak obtained from slopes of curves in a).

Next, we estimated the speed with which the pre-exposed animals reached their destination (near the peak concentration where the worms were paralyzed by Sodium Azide) and compared it with that of control animals by plotting the average distance between the worms and the concentration peak as a function of time in Fig. 8a. The purpose of this analysis is to distinguish between the behavior of *C. elegans* in an environment with and without the gradient of chemoattractant concentration. In absence of food, pre-exposed *C. elegans* predominantly moved in circles and the net displacement was significantly smaller than that of control animals as described earlier. In presence of a chemoattractant concentration gradient, we clearly note that the time taken by pre-exposed worms to reach their destination is the shortest meaning that they tend to move more effectively and reach the chemoattractant more quickly. The speed with which animals approached the peak concentration was then estimated by calculating the slopes of these curves (Fig. 8b). The negative slopes indicate that the worms moved towards the peak concentration. The speed with which pre-exposed worms migrated to the peak concentration immediately after pre-exposure was found to be at least four times that of control animals.

**Figure 9:**
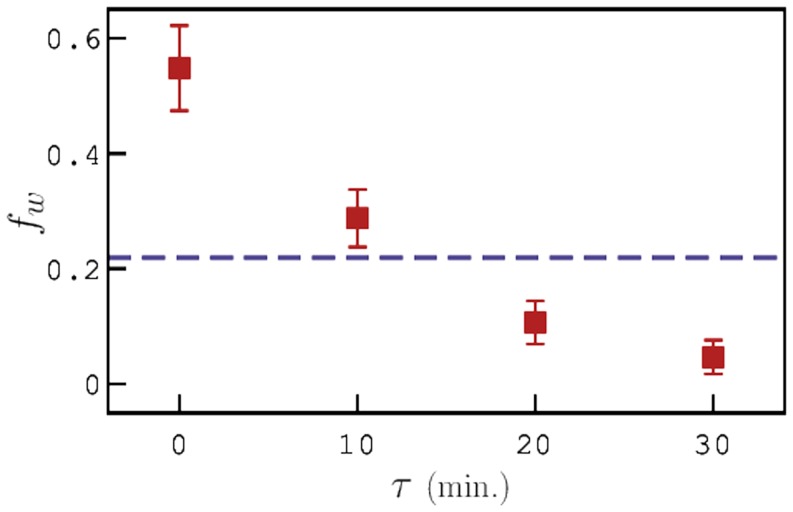
Fraction of worms traveling towards the concentration peak during chemotaxis as a function of recovery time. The dashed-line is for control worms.

As the worms were allowed to recover from the high-temperature shock, the time taken by the animals to reach their destination increased and for worms that were allowed to recover for a period of 30 minutes, we note that the average number of worms never reached their destination indicating that these animals have either lost their sense of chemotaxis or do not have enough energy to crawl towards the peak concentration. We also observed that at these conditions, several worms showed negative chemotaxis. Fig. 9 shows the fraction of worms that traveled successfully towards the peak concentration, calculated as the ratio of number of worms paralyzed near the peak to the total worm population on the plate. We find that, immediately after pre-exposure almost all the worms traveled towards the peak indicating a heightened sense of chemotaxis, whereas after a certain period of recovery, the fraction of worms going in the desired direction drastically dropped indicating a reduction in their chemotaxis ability.

## Discussion

We have thoroughly investigated the effect of temperature pre-exposure on *C. elegans*' behavior, locomotion, and chemotaxis. First, we classified the shapes of *C. elegans* using our recently developed model. We note that for the ratio of parameters 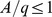, the worms exhibit ‘regular’ sine-shaped structures, while for 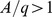, they assume more ‘complex’ shapes including omegas and loops. When *C. elegans* were exposed to high temperatures, we observed a drastic change in their behavior and the probability of animals assuming ‘complex’ shapes was significantly higher than that of control worms that were maintained at the culture temperature of 20*°*C. Some earlier studies on *C. elegans* [34] have indicated that higher temperatures increase their metabolic activity. This suggests that when the worm's metabolic activity is high, which in fact makes them more active, they tend to assume complex shapes more frequently. As the worms recovered from the high-temperature shock, their shapes became ‘normal’ and after a period of 30 minutes after pre-exposure, 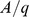 dropped below that of control animals. This could probably be due to hyper-activity during the recovery period immediately after pre-exposure and lack of energy resulting from a drastic drop in the worm's metabolism during the recovery period.

Next, we looked into the effect of temperature pre-exposure on the locomotion of *C. elegans*. The increased level of activity in *C. elegans* immediately after pre-exposure is readily quantified by comparing the distance traveled by both pre-exposed and control worms in a given time, As expected, the pre-exposed worms traveled longer distances with higher velocities compared to control animals. However, the net displacement of pre-exposed worms from the starting points is significantly smaller than that of control animals. To understand this, we analyzed the locomotory behavior of both sets and observed that although the instantaneous velocity of pre-exposed worms is higher, they made more turns than the control animals that made them move around the same spot. This is in agreement with our earlier observations of the shapes. The contour length of the pre-exposed worm trajectory was higher. Whereas, the control animals, although moved slowly, made larger displacements as they spent most of their time moving in forward direction with only occasional turns. Here, we also note that pre-exposure had no significant effect on reversals.

The chemotaxis index of pre-exposed animals showed a steep increase immediately after pre-exposure and gradually decreased as the worms were allowed to recover at room temperature until it fell below that of control animals after 30 minutes of recovery. In absence of food, we observed that *C. elegans* made more turns and did not cover a large area but, in presence of a chemoattractant gradient, we noticed a significant shift in their behavior. The pre-exposed animals approached the peak concentration approximately 4 times faster than the control animals until they reached a point where they got paralyzed by Sodium Azide. This suggests that *C. elegans* are able to effectively manifest high levels of activity by increasing their sense of chemotaxis and navigating in the desired direction.

## Materials and Methods

### Strain culture and staging

Wild-type N2 *C. elegans* were cultured on a 60 × 15 mm petri dish containing 2 *wt*.% agar with *Escherichia coli* OP50 as food source in an incubator maintained at 20*°*C. The petri dish was subsequently wrapped with paraffin film to prevent growth of other bacteria or mold. All experiments were conducted using synchronously staged worm population prepared using hypochlorite digestive treatment [35]. Well-fed young adults (3–4 days old after staging) were selected for all experiments to exclude the possible effects on animal physiology from starvation [36]. To conduct experiments, 1.0 ml of distilled water was poured on to a plate of staged worms and gently swirled for 10 seconds to wash the worms off the plate. The water containing the worms was then transferred to a 15 ml centrifuge tube and the worms were allowed to settle to the bottom of the tube. The worms were washed with fresh distilled water 3–4 times to remove bacteria and other food sources. A drop of sediment containing approximately 400–500 worms were transferred to a fresh plate and the excess water was then removed using a tissue paper. The worms were then allowed to crawl on the agar plate before recording images for analysis.

### Pre-exposure to higher temperature

To study the effect of temperature pre-exposure on the locomotion and chemotaxis of *C. elegans*, a staged plate was kept inside an incubator that was maintained at a temperature of 30*°*C for 30 minutes before the start of actual experiments. After pre-exposure to higher temperature, locomotion and chemotaxis experiments on *C. elegans* were conducted in several batches each with different recovery times. The recovery times used in this study range from 0 to 30 minutes with increment of 10 minutes. Here, a recovery time of 0 minutes mean that experiments were performed on *C. elegans* immediately after pre-exposure. To compare the behavior of pre-exposed worms with non pre-exposed worms, control experiments were also done with worms maintained at the culture temperature of 20*°*C.

### Image acquisition and analysis

Images of *C. elegans* crawling on agar plates were taken using a Zeiss Stemi 2000–C stereo microscope mounted with a video camera (640 × 480 pixels with a 7.4 mm^2^ pixel size and an 8-bit mono sensor), and were saved as black and white avi movie files. The microscope was outfitted for bright field illumination from a light source reflected from a flat mirror positioned at an angle of 45 degrees such that the agar plates were illuminated from below. Worms imaged with this apparatus appear as a dark feature on a light background. Individual worm shapes were obtained from the avi movie files by converting the movie to 8-bit black and white tiff images using ImageJ software (http://rsbweb.nih.gov/ij/). The individual images were then analyzed as follows. A greyscale threshold was applied to the images to separate the worm from the background, small non-worm objects were removed from the images based on their size, ‘holes’ in the thresholded worms' bodies were filled and the solid binary images of the worms' bodies were transformed into a skeleton running down the center of the body by applying a morphological thinning operation. Individual data points were collected from the resulting binary skeleton image by determining which pixels had a value equal to 1, collecting their row and column positions, and utilizing these positions as Cartesian (x,y) coordinates. The normalized skeleton data was then used to quantify the behavior of *C. elegans* by fitting the recently developed model [37] (described below) to it and estimating the parameters that define the shape of the worm.

### Chemotaxis assay

Chemotaxis assay plates contained 1 M sodium acetate solution on 2 *wt*.% agar. The pH of the stock solution was adjusted to 7.0 with acetic acid ([5]). 5 *µ*L of the salt solution was spotted on the agar plate (location A in Fig. 1b) 16–17 hours before the start of experiments to let the salt diffuse through agar and obtain a gradient. Shortly before the chemotaxis assay, 1 *µ*L of 1 M Sodium Azide was spotted onto the same position to anaesthetize the animals at the center of the gradient. As a control, Sodium Azide was also spotted at a position approximately 3 cm away from the center of the test compound gradient at location B. For chemotaxis assays of both pre-exposed and control animals, approximately 400 animals were placed equidistant (∼ 3 cm) from the two spots (location C) and the excess water was removed using a tissue paper. Once the animals started crawling freely on the agar surface, the assay plates were left undisturbed for 30 minutes at room temperature. The number of worms at each of the locations A and B (circle of 1.5 cm radius) was determined by manual counting. The chemotaxis index (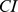) was then calculated as 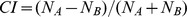 where 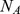 and 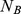 are the number of worms in the two regions A and B, respectively. The 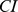 values reported in this work were obtained by averaging over 20 independent chemotaxis assays on both pre-exposed and control animals. For plotting the average distance of worms from the chemoattractant as a function of time to calculate the speed with which the worms approached the peak concentration, their positions were recorded every 10 seconds.

### Piecewise-harmonic-curvature (PHC) model for worm shapes

In our previous work [37], we have developed a simple analytical representation to describe both individual body shapes and entire nematode trails by a continuous line corresponding to a piecewise-harmonic curvature function
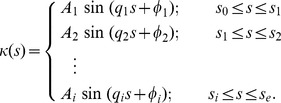
(1)


Here 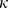 is the local curvature of the worm's body, the parameter 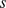 represents the arc length along the trail, 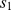, 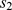,… 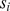 denote the intermediate points of the worm's trail where a sudden transition between harmonic modes occurs, 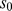 and 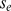 are beginning and end points of the trail, respectively (Fig. 1c), and 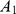, 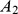,… 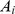 are the amplitudes, 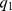, 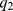,… 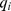 are the wavevectors, and 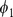, 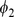,… 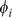 are the phases of the harmonic wave. The shape of the trail or the body posture of the worm can be described by a set of coupled second-order differential equations
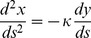
(2a)




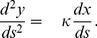
(2b)


Equations (2) are solved with the initial conditions
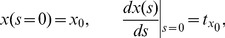
(3a)




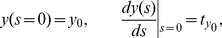
(3b)where 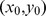 is the position of the worm tail at the beginning of the trail and the tangent unit vector, 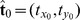 describes the orientation of the tail at *t*  =  0. The curvature-based kinematic model for body shapes of *C. elegans* provides a low-dimensional unified description for the wide spectrum of shapes and body postures the worm assumes during gradual turns and sharp turns/pirouettes for chemotaxis in gradient environments. We refer interested readers to the original article by Padmanabhan *et al.* [37] In this work, we have used this model to quantify the shapes of *C. elegans* by calculating amplitude 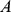 and wave vector 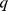 of the worms body curvature during locomotion and chemotaxis.
